# Effects and Mechanisms of Dufulin Toxicity on Zebrafish, *Danio rerio*

**DOI:** 10.3390/toxics13121075

**Published:** 2025-12-13

**Authors:** Shaoqian Jia, Mengxue Li, Guoqiang Yuan, Long Wang, Heng Chi

**Affiliations:** 1 Food and Pharmaceutical Research Institute, Jiangsu Food & Pharmaceutical Science College, Huai’an 223300, China; qian12358@163.com (S.J.); 20231031@jsfpc.edu.cn (M.L.); 2 School of Chemistry and Chemical Engineering, Yangzhou University, Yangzhou 225009, China; 3 School of Life Science, Huaiyin Normal University, Huai’an 223300, China

**Keywords:** dufulin, *Danio rerio*, transcriptome, toxicity

## Abstract

Pesticide pollution has become a major global issue, primarily because of its ability to induce widespread toxicity. This research aimed to explore the toxicological effects of Dufulin on zebrafish, focusing on the underlying molecular mechanisms of these effects from biochemical and transcriptomic perspectives. Residual Dufulin has been confirmed to occur in ecosystems, with its half-life significantly affected by environmental conditions. Its low water solubility may lead to its accumulation in sediments, thereby posing a potential threat to aquatic ecosystems, which necessitates urgent targeted research. The findings of this study indicate that Dufulin has concentration-dependent effects on zebrafish; 0.01 mg/L Dufulin triggered a non-specific immune response and enhanced the antioxidant defense system in zebrafish, resulting in oxidative stress or apoptosis and influencing the cell cycle, while 0.10 mg/L Dufulin mainly affected normal meiosis of zebrafish cells and 1.00 mg/L Dufulin produced cytotoxicity and affected the normal metabolic process of zebrafish. These findings reveal the multi-level toxic mechanism of Dufulin on aquatic organisms from the biochemical and transcriptome levels and provide an important basis for its ecological and environmental risk assessment.

## 1. Introduction

Pesticides play a vital role in agricultural production [1]. With the continuous advancement of agriculture, traditional disease control has been unable to meet the production requirements of high-yield and high-quality crops, so the development of new antiviral pesticides is important. In the past few decades, a variety of highly effective and slightly toxic pesticides have been developed for agricultural protection, such as Dufulin [2,3]. As a new antiviral pesticide, Dufulin significantly improves plants’ resistance to viruses by activating systemic acquired resistance (SAR) [4,5]. Previous studies have shown that Dufulin can inhibit the RNA-silencing suppressor (P6) of viruses and protect plants from viral diseases (such as Southern rice black-streaked dwarf virus) [6,7]. Therefore, China has registered Dufulin as an antiviral pesticide for major crops such as rice, tobacco, and vegetables [5,8,9]. Regarding levels of residual Dufulin in the environment, field studies in Guizhou and Shandong Provinces showed that after applying 30% Dufulin wettable powder at twice the maximum recommended dosage (1000 g.a.i/ha), its residual concentration in soil dropped to below 0.31 mg/kg within 21 days; in laboratory-simulated contaminated soil for benthic organism toxicity tests, the concentration ranged from 10.0 to 911.6 mg/kg (encompassing both racemic Dufulin and its individual enantiomers). In aquatic environments, Dufulin residues were detected in paddy field water post-application, while in a study on oxidative stress metabolomics in Tubifex, the laboratory test concentrations were in the range of 1 × 10^−4^ to 216.9 mg/L [10,11,12].

When sprayed, pesticides may remain on crop surfaces and in soil [13,14]. They may then enter water from the soil through runoff, or enter air from crop surfaces through volatilization or wind erosion, and finally enter rivers through rainfall [14,15,16]. Once these residual pesticides enter a river, they can be deposited on sediments, endangering environmental and human food safety [14,15]. Moreover, pesticide abuse has caused considerable environmental pollution [17]. Pesticides can enter the human body through a variety of pathways, such as breathing, skin contact, and the food chain, which can directly lead to poisoning and even death [18,19].

Previous studies have indicated that Dufulin, as a new type of pesticide, has adverse effects on organisms, and that similarly to various other pesticides and environmental pollutants, it can induce oxidative stress within biological systems [14,20]. This process is characterized by an imbalance between the production and elimination of reactive oxygen species (ROS), leading to an accumulation of these harmful byproducts. The ability of Dufulin to generate substantial quantities of ROS raises concerns about its potential adverse effects on both human health and the environment, mirroring the observed impacts of other chemical agents [14,20,21]. Dufulin may affect the antioxidant defense system of organisms, leading to changes in antioxidant enzyme activities or enzyme structure and a decrease or increase in the activity of antioxidant-related enzymes (superoxide dismutase (SOD), catalase (CAT)) [14,20,21]. Meanwhile, high levels of ROS can inhibit the self-repair of DNA, leading to lipid peroxidation and the production of large amounts of malondialdehyde (MDA), ultimately inducing cell apoptosis and DNA damage [22,23]. Previous studies have indicated that pesticides may have adverse effects on biological functions, such as neurotoxicity, hepatotoxicity, and immune disorders [24,25]. The non-specific immune system in fish is more sensitive to changes in the external environment, especially in alkaline phosphatase (AKP) and acid phosphatase (ACP) [26]. Studies have shown that pesticides can directly interfere with the innate immune system of zebrafish by regulating the expression pattern of immune-related genes such as Il-8 and Tnf-α, thus causing immunotoxicity [27,28]. As an important model organism, zebrafish has become an indispensable research subject in toxicology research and plays a vital role in evaluating the toxicity and molecular mechanisms of chemical substances. Moreover, zebrafish embryos can be used to detect environmental pollutants, providing a scientific basis for environmental protection and human food safety [21,28,29,30].

At present, the research on Dufulin mainly focuses on its effects on plants and determining its environmental levels, while its toxic effects on vertebrates, especially fish, and the potential mechanisms of its toxicity have not yet been reported [9,12,21,31,32]. In order to understand the impact of Dufulin concentration on the immune response and antioxidant defense system of zebrafish, it is necessary to conduct thorough investigations. This study seeks to explore the toxicity levels associated with different doses of Dufulin, examining how these levels affect the overall health and biological functions of zebrafish. Moreover, it aims to uncover potential regulatory mechanisms influenced by Dufulin exposure, with multiple perspectives considered for a more robust analysis. Notably, this marks the first attempt to investigate these effects and their mechanisms in zebrafish, highlighting the significance and novelty of this research. The results provide baseline data on the toxic effects of pesticides on fish and other vertebrates, establishing a basis for evaluating the ecological risks of pesticides (such as Dufulin) to aquatic environments. Furthermore, they offer valuable insights into the toxic mechanisms through which pesticides exert their harmful effects, paving the way for a deeper exploration of the relationship between pesticide exposure and physiological responses in aquatic species. This understanding of toxicity and its molecular mechanism is vital for developing strategies to mitigate the adverse effects of pesticides and protect aquatic ecosystems.

## 2. Materials and Methods

### 2.1. Chemicals and Animals

The chemical Dufulin (95.5% purity) and three-month-old zebrafish (wild-type, AB strain) were provided by the Jiangsu Food and Pharmaceutical Science College. The zebrafish were reared in a cultivation system specifically designed for them (maintained at 25 ± 1 °C, with a pH of 7.5 and dissolved oxygen levels of at least 5.5 mg/L, following a 14 h light and 10 h dark cycle) for a period of two weeks, and were fed brine shrimp twice each day [33]. Uneaten food and feces were extracted using a siphon. This study was approved by the Animal Care and Use Committee of the Jiangsu Food and Pharmaceutical Science College (approval number: JFPSC20241010).

### 2.2. Experimental Design

After two weeks, healthy zebrafish were selected and placed in plastic tanks (40 × 20 × 20 cm^3^). Each tank contained 4 L water. The water conditions in the tank were as follows: standard dilution water; 25 ± 1 °C; pH = 7.5; DO ≥ 5.5 mg/L [33]. The concentrations of Dufulin were chosen according to environmentally relevant levels and the methodology of a previous study [14]. The Dufulin concentrations were set as follows: 0, 0.01, 0.1, and 1 mg L-1. Fifteen healthy zebrafish were assigned to each concentration group, and the experiment was repeated three times in each group (a total of 180 zebrafish). To ensure acceptable water quality and Dufulin concentration, 1/2 of the water was renewed daily, and then more Dufulin was added to the water. The exposure experiment lasted 21 days.

### 2.3. Biochemical Analysis

Following the exposure experiment, the zebrafish were put on ice. When the zebrafish were stationary, sterile scissors and forceps were used to remove tissue, specifically the liver. We accurately weighed the liver and added 0.8% saline (9:1/buffer volume: zebrafish tissue weight), then ground the tissue on ice; finally, a tissue homogenate was obtained. The tissue homogenate was subjected to centrifugation at 4 °C for 10 min at 2500 rpm, and the resulting supernatant was then utilized for biochemical analysis. First, the protein concentration in the liver was determined using the BCA method with a multifunctional microplate reader (SpectraMax ID5, BioTek, Winooski, VT, USA) at a wavelength of 562 nm. Then, biochemical indicators in the liver were measured according to the instructions for commercial test kits (provided by Beijing Solarbio Science & Technology Co., Ltd., Beijing, China). The specific methods were performed following our protocol, which is provided in the Supplementary Materials. Malondialdehyde (MDA) content was determined to be 532 nm. Superoxide dismutase (SOD) activity was represented by a wavelength of 450 nm; catalase (CAT) activity by a wavelength of 520 nm; peroxidase (POD) activity by a wavelength of 470 nm; alkaline phosphatase (AKP) activity by a wavelength of 405 nm; and acid phosphatase (ACP) activity by a wavelength of 405 nm. The concentration of protein in the liver was determined to be 562 nm.

### 2.4. RNA Sequencing Analysis

A single sample comprised three zebrafish livers, and each concentration group underwent three experimental repetitions, resulting in 12 samples being utilized for RNA sequencing analysis. Total RNA from the zebrafish livers was extracted following the instructions of a commercial assay kit provided by Beijing Solarbio Science & Technology Co., Ltd., China. Following the quality evaluation, the extracted RNA underwent further experiments, and Genedenovo Biotechnology Co., Ltd. (Guangzhou, China) was employed for the construction of the cDNA library. Sequencing was performed using the Illumina Novaseq6000 platform (Genedenovo Biotechnology Co., Ltd. (Guangzhou, China)), and then an analysis of the transcriptome data was conducted. DESeq2 (v 1.48.1) software as crucial in conducting the differential expression analysis as it allowed for the identification of differentially expressed genes (DEGs), which were determined using a stringent criterion that included a false discovery rate (FDR) threshold of 0.05 or lower, combined with the requirement of an absolute fold change of 2 or greater. Following the identification of these DEGs, further analyses were carried out to explore their biological significance. Specifically, both Gene Ontology (GO) enrichment analysis and Kyoto Encyclopedia of Genes and Genomes (KEGG) enrichment analysis were performed to gain insights into the functional roles and pathways associated with the identified genes.

### 2.5. qRT-PCR Analysis

The precision and dependability of the transcriptome data acquired in this research were confirmed; thus, the 10 differentially expressed genes (DEGs) chosen were utilized for validation. The zebrafish-specific primers for the relevant genes were designed using the NCBI Primer-Blast online tool (https://www.ncbi.nlm.nih.gov/tools/primer-blast/, accessed on 27 January 2025) and supplied by Sangon Biotech (Shanghai) Co., Ltd., Shanghai, China. Their sequences, lengths, and accession numbers are provided in Table 1. The cDNA was reverse-transcribed according to the manual for the commercial assay kit from Beijing Solarbio Science & Technology Co. Ltd., China, and qRT-PCR was conducted according to the manual for the commercial assay kit from Novoprotein Scientific Inc., Suzhou, China. The relative expression levels were calculated according to the 2^−ΔΔCT^ method, and β-actin was employed as the reference gene in this study. The experiment was repeated in each concentration group three times. The specific methods were performed following our protocol, which is provided in the Supplementary Materials.

### 2.6. Statistical Analysis

The transcriptome data obtained in this research are displayed as the mean ± standard deviation (mean ± SD), and data analysis was carried out utilizing SPSS Version 26.0. To assess the differences among various groups, a one-way ANOVA with Duncan’s multiple comparisons was performed. A *p*-value of less than 0.05 indicates statistical significance, whereas a *p*-value greater than 0.05 indicates statistical insignificance.

## 3. Results

### 3.1. Impacts of Dufulin on Biochemical Analysis of Zebrafish

**Figure 1:**
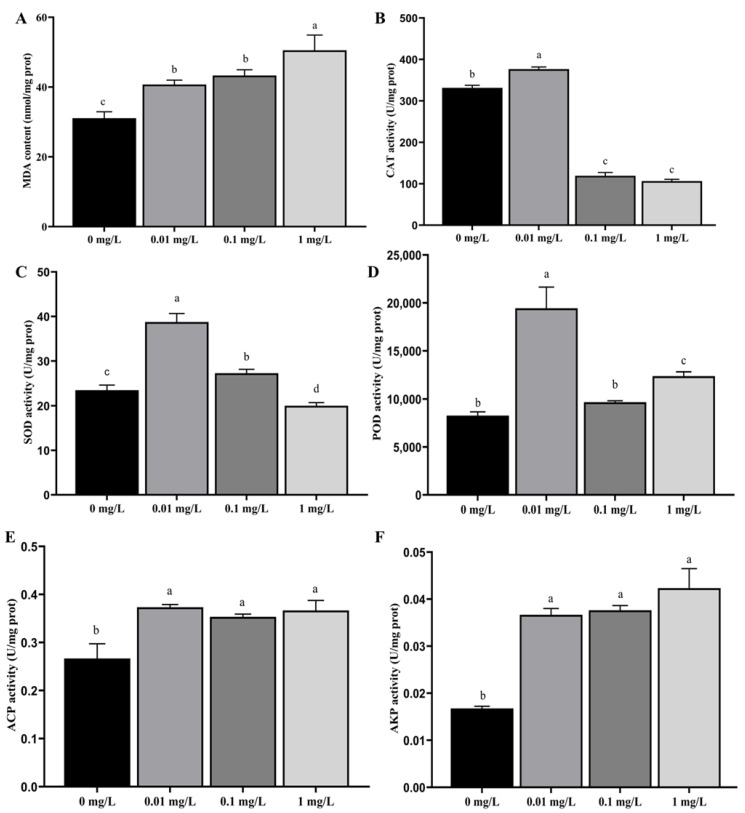
Effects of Dufulin on biochemical parameters in zebrafish, including (**A**) MDA content; (**B**) CAT activity; (**C**) SOD activity; (**D**) POD activity; (**E**) ACP activity; and (**F**) AKP activity. The data are presented as the mean ± SD with three replicates per group. Different letters indicate that there are significant differences between treatments (*p* < 0.05).

As the concentration of Dufulin increased, both MDA content (Figure 1A) and AKP activity (Figure 1F) increased. There were significant differences in MDA content and AKP activity between the control group (0 mg/L) and all treatment groups (*p* < 0.05). As the concentration of Dufulin increased, CAT activity (Figure 1B) and SOD activity (Figure 1C) first increased and then decreased. There were significant differences in CAT and SOD activity between the control group (0 mg/L) and all treatment groups (*p* < 0.05). No meaningful difference in POD activity was noted between the control group (0 mg/L) and the 0.10 mg/L treatment group (*p* > 0.05). As the concentration of Dufulin increased, POD activity (Figure 1D) and ACP activity (Figure 1E) showed a trend of first increasing, then decreasing, and finally increasing. The difference in POD activity between the control group (0 mg/L) and the 0.01 and 1.00 mg/L treatment groups was statistically significant (*p* < 0.05). When comparing the control group (0 mg/L) with the 0.01, 0.10, and 1.00 mg/L treatment groups, significant differences in ACP activity were observed (*p* < 0.05).

### 3.2. Transcriptome Sequencing Data of Zebrafish Exposed to Dufulin Stress

Table 2 presents information on the utilization of 12 samples for RNA sequencing. Each sample produced approximately 5.86 Gb of data, forming a significant dataset for further analysis. The raw sequencing reads ranged from 5.40 × 10^9^ bp to 6.36 × 10^9^ bp, and all samples achieved Q20 scores exceeding 97.73% and Q30 scores exceeding 93.77%. The results derived from the Pearson correlation coefficient clearly indicate a strong degree of consistency among the samples analyzed, which is visually represented in Figure S1. This high level of correlation suggests that the samples are comparable and sufficiently reliable for further investigation. As a result, the dataset is suitable for conducting transcriptome analysis, ensuring that the findings will be based on accurate and reliable data.

**Table 2:** Statistics for the transcriptome data.

Sample	Raw Data (bp)	Clean Data (bp)	Q20 (%)	Q30 (%)	GC (%)
A-1	5.45 × 10^9^	5.26 × 10^9^	98.06%	94.43%	46.78%
A-2	6.08 × 10^9^	5.80 × 10^9^	98.12%	94.55%	47.31%
A-3	5.52 × 10^9^	5.33 × 10^9^	97.91%	94.16%	48.17%
B-1	6.36 × 10^9^	6.17 × 10^9^	97.87%	94.07%	46.52%
B-2	6.33 × 10^9^	6.13 × 10^9^	97.91%	94.16%	46.14%
B-3	5.43 × 10^9^	5.23 × 10^9^	97.92%	94.11%	46.95%
C-1	6.14 × 10^9^	5.94 × 10^9^	97.88%	94.07%	47.01%
C-2	5.49 × 10^9^	5.31 × 10^9^	98.04%	94.46%	48.14%
C-3	5.95 × 10^9^	5.78 × 10^9^	97.93%	94.18%	47.63%
D-1	6.01 × 10^9^	5.78 × 10^9^	97.97%	94.29%	46.55%
D-2	5.40 × 10^9^	5.19 × 10^9^	97.73%	93.77%	47.34%
D-3	6.18 × 10^9^	5.95 × 10^9^	97.97%	94.25%	47.47%

Note: A represents the 0 mg/L treatment group; B represents the 0.01 mg/L treatment group; C represents the 0.10 mg/L treatment group; D represents the 1.00 mg/L treatment group.

### 3.3. Analysis of Differentially Expressed Genes

**Figure 2:**
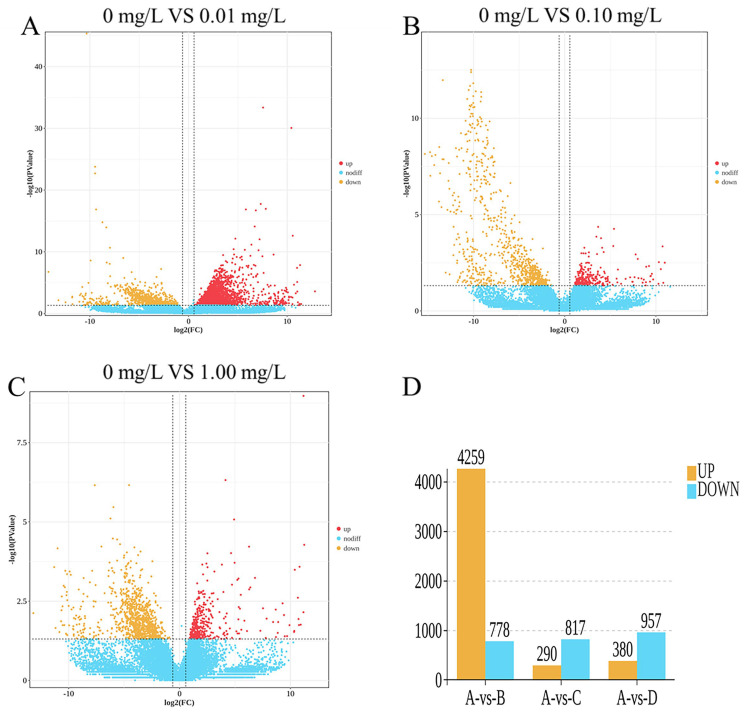
Differentially expressed gene (DEG) statistics: (**A**) 0 mg/L vs. 0.01 mg/L; (**B**) 0 mg/L vs. 0.10 mg/L; (**C**) 0 mg/L vs. 1.00 mg/L; (**D**) Venn diagram of DEGs. Red dots represent significantly upregulated DEGs, yellow dots represent downregulated DEGs, and blue dots represent DEGs with no significant difference in expression.

The results of the statistical analysis of differentially expressed genes (DEGs) are illustrated in Figure 2. A total of 5962 DEGs were identified.

As shown in Figure 2A,D, the 0.01 mg/L treatment group exhibited 5037 differentially expressed genes (DEGs), which included 4259 upregulated genes and 778 downregulated genes. As shown in Figure 2B,D, the 0.10 mg/L treatment group exhibited 1107 differentially expressed genes (DEGs), which included 290 upregulated genes and 817 downregulated genes. As shown in Figure 2C,D, the 1.00 mg/L treatment group exhibited 1337 differentially expressed genes (DEGs), which included 380 upregulated genes and 957 downregulated genes.

**Figure 3:**
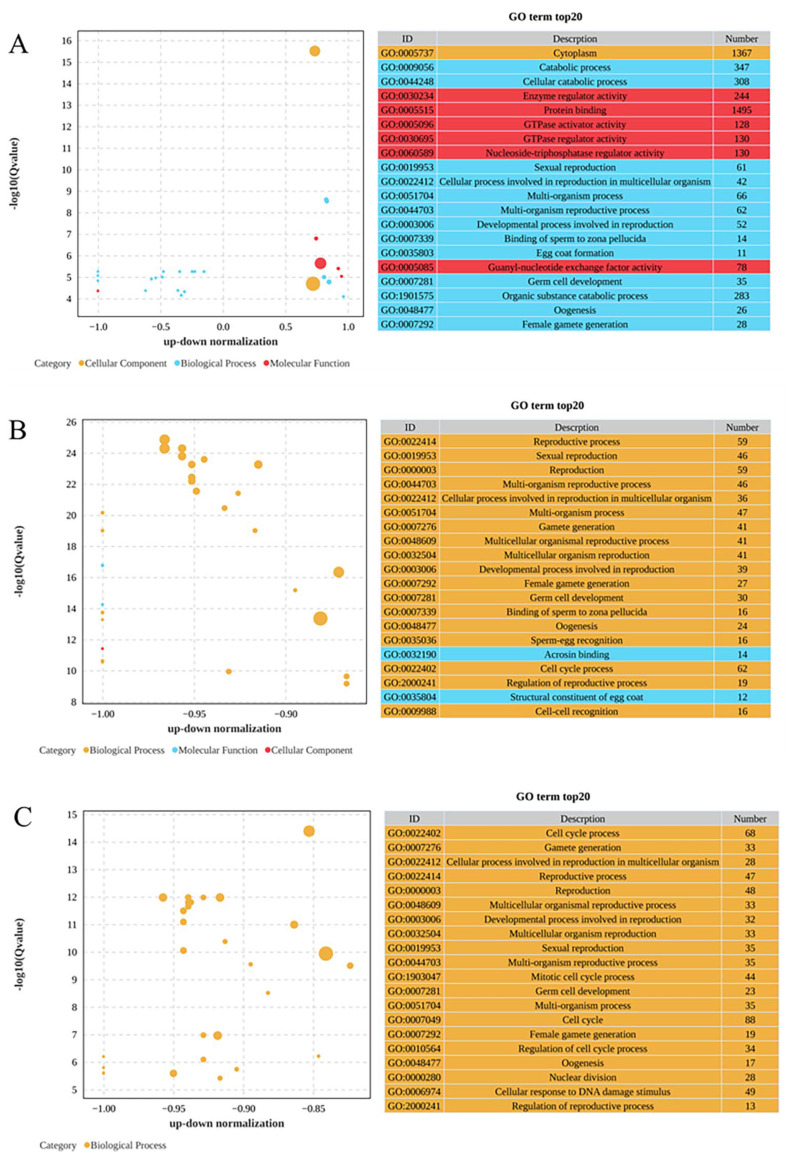
Gene Ontology (GO) enrichment analysis of DEGs: (**A**) 0 mg/L vs. 0.01 mg/L; (**B**) 0 mg/L vs. 0.10 mg/L; (**C**) 0 mg/L vs. 1.00 mg/L. Bubble plot showing the 20 most enriched GO terms in the biological process (blue), molecular function (orange) and cell component (red) categories. The −log10 (Q value) is shown in the *Y*-axis, and the z-score is shown in the *X*-axis.

As demonstrated in Figure 3, the GO enrichment analysis organized these DEGs into three main categories: biological processes, cellular components, and molecular functions. In the comparison between the 0 mg/L and 0.01 mg/L treatments, as depicted in Figure 3A, the biological process category showed significant enrichment for the catabolic process (GO:0009056), followed closely by the cellular catabolic process (GO:0044248) and sexual reproduction (GO:0019953). In terms of cellular components, cytoplasm (GO:0005737) was significantly enriched, while enzyme regulatory activity (GO:0030234) was most significantly enriched in the molecular functions category, followed by protein binding (GO:0005515) and GTPase activator activity (GO:0005096). Among these GO terms, most DEGs were upregulated in the 0.01 mg/L treatment group. As depicted in Figure 3B, the comparison of the 0 mg/L and 0.10 mg/L treatments revealed that the reproductive process (GO:0022414) was the most significantly enriched under the biological processes, accompanied by significant enrichment in sexual reproduction (GO:0019953) and reproduction (GO:0000003). Among the molecular functions, acrosin binding (GO:0032190) exhibited the most significant enrichment, followed by the structural constituent of the egg coat (GO:0035804). Furthermore, as presented in Figure 3C, the comparison of the 0 mg/L and 1.00 mg/L treatments highlighted significant enrichment in the cell cycle process (GO:0022402) in the biological process category, as well as in gamete generation (GO:0007276) and cellular processes related to reproduction in multicellular organisms (GO:0022412).

**Figure 4:**
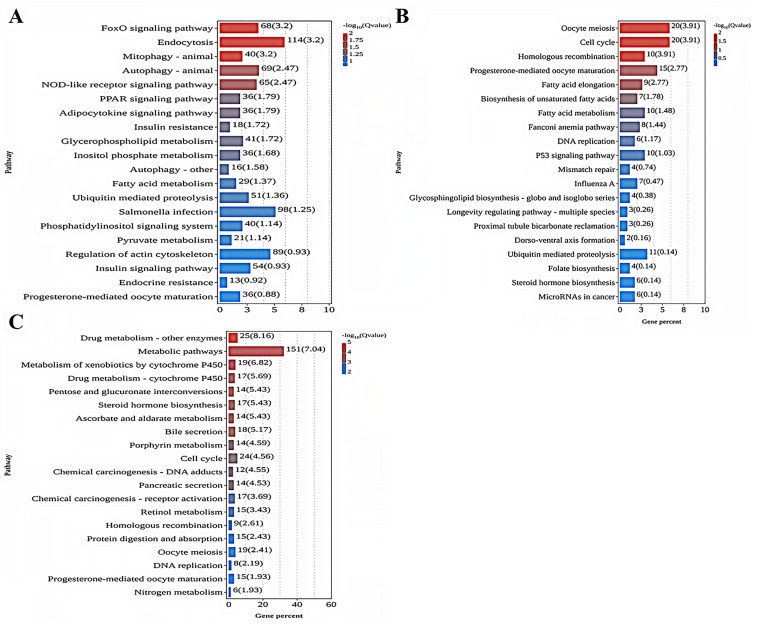
KEGG pathway enrichment analysis of DEGs in each comparison group: (**A**) 0 mg/L vs. 0.01 mg/L; (**B**) 0 mg/L vs. 0.10 mg/L; (**C**) 0 mg/L vs. 1.00 mg/L. The *X*-axis denotes the percentage of genes in this pathway accounting for the total number of annotated genes. The *Y*-axis represents the name of the pathway.

As shown in Figure 4A, the analysis of KEGG enrichment indicated that the five most enriched KEGG pathways in the comparison between the 0 mg/L and 0.01 mg/L treatment groups were the FoxO signaling pathway (ko04068), Endocytosis (ko04144), Mitophagy—animal (ko04137), Autophagy—animal (ko04140), and the NOD-like receptor signaling pathway (ko04621). Among these KEGG terms, most DEGs were found to be upregulated in the 0.01 mg/L treatment group. As shown in Figure 4B, the comparison between the 0 mg/L and 0.10 mg/L treatment groups indicated that the most enriched pathways were Oocyte meiosis (ko04114), Cell cycle (ko04110), Homologous recombination (ko03440), Progesterone-mediated oocyte maturation (ko04914), and Fatty acid elongation (ko00062). For these KEGG terms, most DEGs were downregulated in the 0.10 mg/L treatment group. Lastly, as depicted in Figure 4C, the comparison of the 0 mg/L with 1.00 mg/L treatment groups showed that the pathways with the greatest involvement were Drug metabolism—other enzymes (ko00983), Metabolic pathways (ko01100), Metabolism of xenobiotics by cytochrome P450 (ko00980), Drug metabolism—cytochrome P450 (ko04914), and Pentose and glucuronate interconversions (ko00040). In this case, most DEGs were upregulated in the 1.00 mg/L treatment group.

### 3.4. qRT-PCR Validation

**Figure 5:**
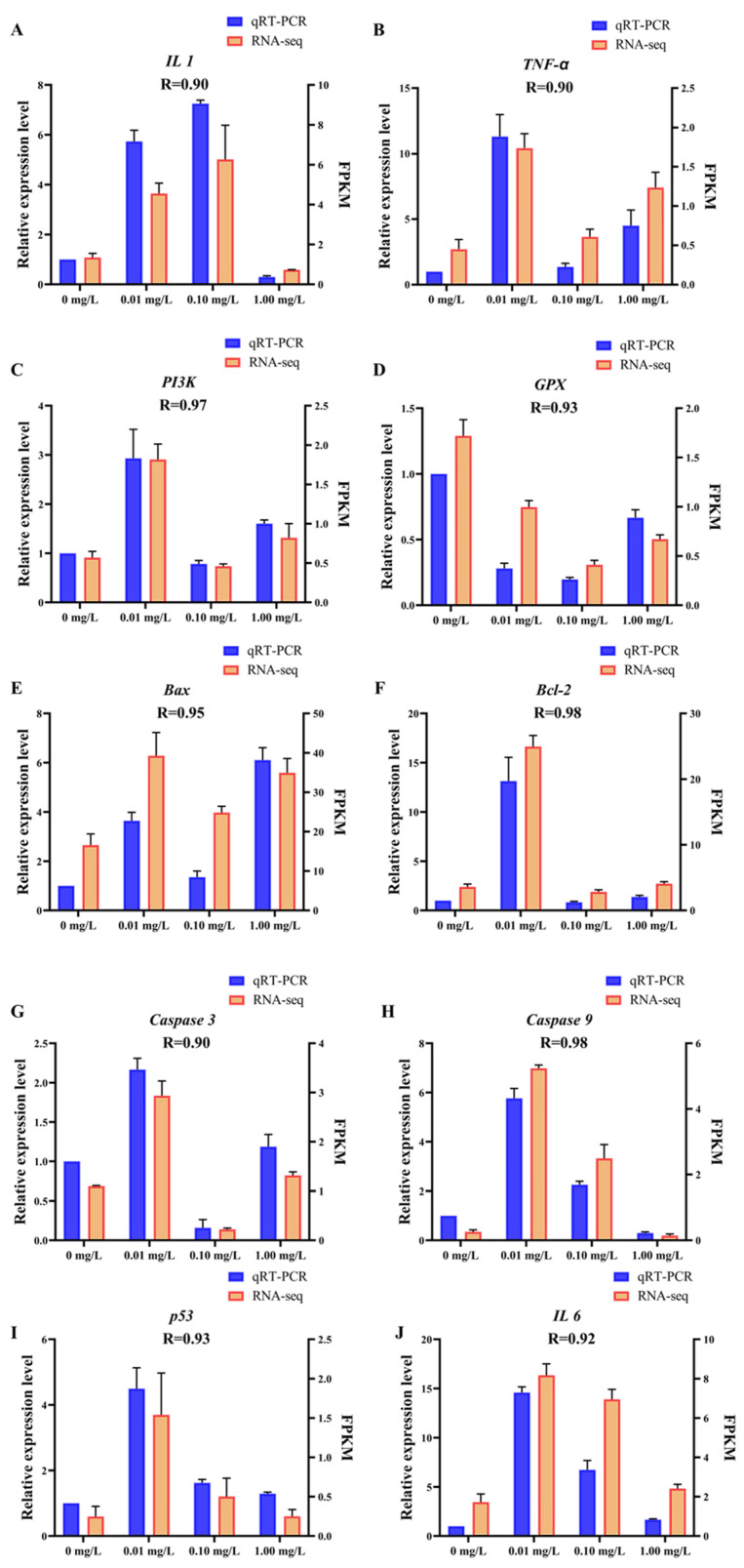
qRT-PCR validation. The *X*-axis represents concentrations of Dufulin, the left *Y*-axis represents the relative expression levels of related genes according to qRT-PCR, and the right *Y*-axis represents FPKM according to RNA-Seq. The qRT-PCR data are expressed as the mean ± SE (*n* = 3). (**A**) *IL 1*, (**B**) *TNF-α*, (**C**) *PI3K*, (**D**) *GPX*, (**E**) *Bax*, (**F**) *Bcl-2*, (**G**) *Caspase 3*, (**H**) *Caspase 9*, (**I**) *p53*, (**J**) *IL 6*.

The data presented in Figure 5 demonstrate a strong relationship between the results obtained from RNA-Seq and qRT-PCR. Specifically, the correlation coefficient (R) values ranged from 0.90 to 0.98. These findings suggest that the transcriptome sequencing data generated in this research is precise and reliable, affirming the validity of the results and the methodology employed in the research.

## 4. Discussion

This research examined the impacts of Dufulin at varying concentrations on the biochemical indicators in zebrafish. Previous studies have indicated that Dufulin, similarly to other pesticides, can influence the antioxidant and immune defense mechanisms of living organisms [14,22,34], underscoring the importance of understanding how exposure to such compounds can compromise the biological systems of aquatic life, particularly during critical developmental stages [14,22,34]. Pesticides may trigger oxidative stress and result in lipid peroxidation by elevating the levels of reactive oxygen species (ROS). Consequently, malondialdehyde (MDA) emerges as a common marker for lipid peroxidation, as it is a final byproduct of this process [23]. The antioxidant system in living organisms primarily depends on the enzyme system responsible for antioxidants (including SOD, CAT, and POD), and the activity levels of these enzymes serve as key indicators for assessing the performance of the antioxidant system [23]. This study found that as the concentration of Dufulin increased, the MDA content showed a gradual upward trend, while the antioxidant enzyme activity generally showed a trend of first increasing and then decreasing. This indicates that Dufulin can induce oxidative stress in zebrafish and lead to lipid peroxidation. To mitigate the oxidative stress caused by Dufulin, zebrafish can activate their antioxidant defense mechanisms. This biological response involves a concerted effort to enhance the activity of specific enzymes involved in combating oxidative stress. By doing so, the zebrafish can eliminate any surplus reactive oxygen species and threaten their cellular integrity. This adaptive response helps to protect the cells from oxidative damage, thereby preventing cell death that could arise from the toxic effects of oxidative stress. As the concentration of Dufulin continues to increase, the oxidative stress generated exceeds the clearance capacity, and the relevant enzyme activity structure may be destroyed, thereby reducing the activity of antioxidant enzymes; this is in line with the results of previous studies [14,35]. It has been found that POD activity has a nonlinear effect, indicating that high concentrations of Dufulin may produce unconventional toxicity [36]. In addition, alkaline phosphatase (AKP) and acid phosphatase (ACP) play an important role in the nonspecific immune system of vertebrates [35]. The findings of the present study showed that as the concentration of Dufulin increased, the ACP activity and AKP activity gradually increased, indicating that Dufulin has a positive effect on the nonspecific immune system of zebrafish and can activate or enhance the nonspecific immunity of zebrafish, which aligns with the results of previous studies [35,37]. In conclusion, it has been observed that Dufulin can induce oxidative stress in zebrafish, even when present at low concentrations, eliciting a response from the antioxidant and nonspecific immune defense systems in these organisms. However, when the concentration of Dufulin increases significantly, it has detrimental effects on the antioxidant defense mechanisms of zebrafish. This disruption can inhibit or completely compromise the normal functioning of these critical biological systems, leading to oxidative damage within the cells and ultimately triggering the process of cell apoptosis.

In order to gain a deeper understanding of the underlying molecular mechanisms of Dufulin toxicity, this research focused on examining its effects on zebrafish at different concentrations, utilizing a transcriptomic approach to uncover the intricate biological processes involved. This investigation aimed to shed light on how varying levels of Dufulin may affect gene expression and overall development in zebrafish, providing valuable insights into its molecular interactions. In the comparison between the 0 mg/L and 0.01 mg/L treatment groups, the Forkhead box O (FoxO) signaling pathway emerged as the most significantly enriched pathway, as illustrated in Figure S2 and Table 3. Research has indicated that the FoxO signaling pathway is crucial for managing oxidative stress and regulating apoptosis. Additionally, this pathway exerts a notable inhibitory effect on cell proliferation, highlighting its importance in cellular processes [38]. Moreover, the FoxO signaling pathway plays an important role in the resistance of zebrafish to external environmental pollutants [39], and key genes in this pathway may be related to the resistance mechanism. The findings of the present study indicate that the significant majority of differentially expressed genes (DEGs) in the 0.01 mg/L treatment group were upregulated. This observation suggests a potential association between these genes and the response mechanisms employed by zebrafish when subjected to low concentrations of Dufulin stress. Furthermore, these results imply that crucial biological processes such as oxidative stress responses, apoptosis, and cell proliferation in zebrafish may be influenced by this environmental stressor. Previous studies have shown that PI3K can phosphorylate AKT by binding to PDK1, thereby activating AKT, and AKT can protect cells from various stress-induced changes and mediate related metabolic effects, thereby ensuring cell survival. Moreover, AKT can phosphorylate FOXO, causing FOXO to remain in the cytoplasm, thus affecting cell proliferation and apoptosis [40,41]. The findings of this research indicated that the genes AKT, PI3K, PDK1, and FOXO were significantly upregulated in the 0.01 mg/L treatment group, indicating that zebrafish may respond to the oxidative stress, apoptosis, and cell proliferation induced by Dufulin by regulating the expression patterns of these genes. In summary, low concentrations of Dufulin could induce oxidative stress and apoptosis in zebrafish, and zebrafish may reduce oxidative stress by regulating the expression levels of important genes in the FOXO signaling pathway. This can reduce Dufulin toxicity and inhibit abnormal cell proliferation, thereby ensuring cell survival.

The Oocyte meiosis signaling pathway emerged as the most notably enriched pathway in the 0 mg/L treatment group compared to the 0.10 mg/L treatment group (Figure S3 and Table 3). This is notable, as previous research has shown that the Oocyte meiosis signaling pathway is crucial for cell meiosis and oocyte maturation [42]. The findings of this research showed that most DEGs in the 0.10 mg/L treatment group were downregulated, indicating that, in the Oocyte meiosis signaling pathway, these genes may be related to the response mechanism of zebrafish to Dufulin stress, and cell meiosis and oocyte maturation may be affected. Phosphorylation of CPE-binding protein (CPEB) is essential for meiosis [43], and during the development of vertebrate oocytes, newly born oocytes must enter meiosis I (MI), thus transitioning from meiosis I to meiosis II (MII). As an important component of maturation-promoting factors, Cdc2 is a crucial driver of the normal progress of oocyte meiosis [44], while Emi is an important regulator of changes in the state of cell meiosis [45]. The results of the present study showed that the genes CPEB, Cdc2, and Emi1 were significantly downregulated in the 0.10 mg/L treatment group, indicating that 0.10 mg of Dufulin can disrupt the meiosis of zebrafish oocytes by inducing changes in the expression pattern of key genes in the Oocyte meiosis signaling pathway.

In the comparison between the 0 mg/L and 1.00 mg/L treatment groups, the pathway that displayed the highest level of enrichment was the Drug metabolism signaling pathway, as illustrated in Figure S4 and Table 3. A previous study has shown that the Drug metabolism signaling pathway is crucial to the metabolic process in organisms, and plays an important role in regulating the oxidation, reduction, hydrolysis, and combination of substances in organisms [46]. The findings of the present study indicated that the significant majority of differentially expressed genes (DEGs) in the treatment group exposed to 1.00 mg/L of Dufulin were upregulated. This pattern suggests that these genes play a critical role in the response mechanisms of zebrafish when subjected to high concentrations of Dufulin. Furthermore, it implies that the metabolic system of zebrafish may experience alterations as a result of this stressor, highlighting the potential impact of Dufulin exposure on their biological processes. The expression of the thiopurine S-methyltransferase (TPMT) gene is often used to evaluate cytotoxicity. As an important enzyme in the metabolism of azathioprine drugs, the activity of TPMT can directly affect the degree of poisoning in an organism. Low TPMT enzyme activity can lead to excessive concentrations of the metabolite 6-thioguanine nucleotide (6-TGN), which has obvious cytotoxic effects [47]. Carboxylesterase (CES, E.C.3.1.1.1) is an important member of the serine hydrolase superfamily, and can catalyze ester- or amide-containing substrates to produce corresponding alcohols and carboxylic acids, thus playing a key role in detoxification. The findings of the present study revealed that certain genes, including TPMT and CES1, were significantly upregulated in the 1.00 mg/L treatment group. This indicates that 1.00 mg of Dufulin has a considerable impact on the metabolic processes occurring in zebrafish. Furthermore, the stress induced by high concentrations of Dufulin appears to elicit cytotoxic effects. In response to this, zebrafish may activate specific metabolic signaling pathways that lead to the production of protective substances. This adaptive response is crucial for mitigating the adverse effects of cytotoxicity and ensuring the survival of the cells involved.

In addition to the previously discussed genes and signaling pathways, this study has uncovered further significant findings, as illustrated in Table 3. The expression levels of the identified genes, along with the associated pathways, are substantially correlated with how zebrafish respond to stress induced by Dufulin. These results enhance our comprehension of the impacts of pesticides on living organisms, as well as the intricate molecular mechanisms underlying their toxic effects.

**Table 3:** DEGs in the most significantly enriched pathway in each group.

Pathway/Gene ID	Gene Name	log2 (Fold Change)
FoxO signaling pathway in 0 mg/L vs. 0.01 mg/L group		
ENSDARG00000075456	*PI3K*	1.8
ENSDARG00000062304	*PDK1*	2.5
ENSDARG00000099657	*Akt*	5.3
ENSDARG00000099555	*FOXO*	1.9
ENSDARG00000036096	*Smad3*	1.7
ENSDARG00000022712	*STAT3*	1.9
ENSDARG00000035557	*ATG8*	1.5
ENSDARG00000075282	*IRS*	3.7
ENSDARG00000076554	*p21*	4.6
ENSDARG00000099719	*p21*	1.7
ENSDARG00000061108	*CPB*	1.8
Oocyte meiosis in 0 mg/L vs. 0.10 mg/L group		
ENSDARG00000008454	*CPEB*	−10.7
ENSDARG00000057328	*C33-4*	−14.3
ENSDARG00000087554	*Cdc2*	−3.4
ENSDARG00000039020	*Emi1*	−5.5
ENSDARG00000004713	*Mad2*	−5.1
ENSDARG00000059131	*Ringo*	−5.5
Drug metabolism in 0 mg/L vs. 1.00 mg/L group		
ENSDARG00000028012	*TPMT*	2.9
ENSDARG00000104818	*CES1*	1.4
ENSDARG0000001726	*CES2*	1.7
ENSDARG00000110614	*UGT*	4.0
ENSDARG00000011521	*UPB1*	1.2
ENSDARG00000079543	*DPYS*	1.3
ENSDARG00000033364	*GST*	1.8
ENSDARG00000017388	*GSTP*	2.5

## 5. Conclusions

This research explored the toxic effects of the pesticide Dufulin on zebrafish and explored the molecular mechanisms of Dufulin toxicity on zebrafish at the biochemical and transcriptomic levels. The results showed that different concentrations of Dufulin led to different molecular mechanisms of toxicity. Briefly, 0.01 mg/L Dufulin can induce oxidative stress, leading to cell apoptosis, and can activate the antioxidant defense system; 0.10 mg/L Dufulin can hinder the meiosis of zebrafish cells; and 1.00 mg/L Dufulin can interfere with the metabolic process of zebrafish and produce cytotoxicity. By establishing a baseline of data, this study serves as a basis for improving our understanding of how pesticides affect various organisms in aquatic environments.

## Figures and Tables

**Table 1:** Primers used in this study.

Primer	Forward and Reverse Primer Sequences (5′-3′)	Length (bp)	Accession Number
*IL1*	F: AAGGCTCCGCTCCACATCTCGTA R: GTCCATCTCCACCATCTGCGAATCT	128	NM 212844.2
*TNF-α*	F: CTCCGAACTTGACGACGAACT R: TTTACTAATTTCAAGCCACC	250	BC124141
*PI3K*	F: CGATGGCAAGTACGGCTTCTCAG R: GCTGGTGCTTGGACACAGGATAC	136	NM_001281844.1
*GPX*	F: GAGGCACAACAGTCAGGGATTA R: AGGAACGCAAACAGAGGGTG	125	NM_001007281
*Bax*	F: GGCTATTTCAACCAGGGTTCC R: TGCGAATCACCAATGCTGT	86	NM_131562.2
*Bcl-2*	F: TCACTCGTTCAGACCCTCAT R: ACGCTTTCCACGCACAT	135	NM_001030253.2
*Caspase 3*	F: ACTGGATCCTGGTGTGGAAACTGA R: CCTGGTCATGATCTGCAAGAGC	129	NM_131877.3
*Caspase 9*	F: GAAGACGGCGAAATCGATGC R: CTGGCGGTTCTGACAACTTCC	248	NM_001007404.2
*p53*	F: GGGCAATCAGCGAGCAAA R: ACTGACCTTCCTGAGTCTCCA	112	NM_001271820.1
*IL6*	F: TCCTGGTGAACGACATCAAA R: TCATCACGCTGGAGAAGTTG	141	NM_001261449.1
*β-actin*	F: CCATCTATGAGGGTTACGC R: GACAATTTCTCTTTCGGCT	86	AF025305.1

## Data Availability

The data will be made available on request.

## Supplementary Materials

The following supporting information can be downloaded at: https://www.mdpi.com/article/10.3390/toxics13121075/s1. Figure S1: The pearson correlation coefficient between samples; Figure S2: The most significantly enriched pathway in 0 mg/L VS 0.01 mg/L: FoxO signaling pathway; Figure S3: The most significantly enriched pathway in 0 mg/L VS 0.01 mg/L: Oocyte meiosis signaling pathway; Figure S4: The most significantly enriched pathway in 0 mg/L VS 0.01 mg/L: Drug metabolism signaling pathway; Supplemental Methods.
