# Supplementary material for: Effects and Mechanisms of Dufulin Toxicity on Zebrafish, Danio rerio
Source: Toxics. 2025 Dec 13;13(12):1075. doi: 10.3390/toxics13121075 (PMC12737344; doi:10.3390/toxics13121075)
Supplement: Supplementary file 1 [file toxics-13-01075-s001.zip › toxics-40368690-supplemental methods-edited.pdf]

Supplemental Methods

Supplemental Methods - Biochemical analysis

1. **MDA content**

First, preheat the microplate reader (Varioskan LUX)) for 30 minutes and adjust to zero with ddH_2O. Follow the detection kit provided by Beijing Solar BioScience & Technology Co., Ltd.

1. Reagent Preparation:

a. Preparation of TBA stock solution: Dissolve an appropriate amount of TBA in TBA Preparation Solution to reach a final concentration of 0.37% (g/ml).

b. Preparation of MDA detection working solution: The MDA assay working solution must be freshly prepared. Please refer to the table below to prepare the appropriate amount of working solution.

Number of Assays	1	10	20	50
TBA Dilution Buffer	150 μL	1500 μL	3000 μL	7500 μL
TBA Stock Solution	50 μL	500 μL	1000 μL	2500 μL
Antioxidants	3 μL	30 μL	60 μL	150 μL

c. Dilution of MDA standards: Dilute the MDA Standard with distilled water to generate 1, 2, 5, 10, 20, and 50 μM standards.

2. Sample Preparation:

Tissue (10 mg) can be homogenized on ice or at 4°C in 0.1 ml of PBS. After homogenization followed by centrifuge at 10,000 × g for 10 minutes, collect the supernatant for MDA assay.

3. Assay Procedure:

a. Add 0.1 mL standard or sample into each vial containing 0.2 mL of MDA detection working solution. PBS used for sample preparation should be used as the blank control to substract the background values from all readings. Refer to the following table to set up the reactions:

	Blank	Standard	Sample
PBS	0.1 mL	—	—
Standard	—	0.1 mL	—
Sample to be tested	—	—	0.1 mL
MDA detection working solution	0.2 mL	0.2 mL	0.2 mL

b. Mix well and incubate the mixture at 100 °C or boiling water for 15 minutes. Care must be taken to avoid liquid bumping and splashing during the incubation. A thermal cycler with a heated lid that can accommodate 0.5 ml PCR tubes is suggested.

c. Cool to room temperature in a water bath and centrifuge at 1000×g for 10 minutes at room temperature. Pipette 200 μl supernatant from each sample into a 96-well plate, and then measure the absorbance at 532 nm with a microplate reader.

d. Calculation of MDA content: the molar concentration of MDA can be determined directly from the standard curve.

2. **SOD activity**

First, preheat the microplate reader (Varioskan LUX)) for 30 minutes and adjust to zero with ddH_2O. Follow the detection kit provided by Beijing Solar BioScience & Technology Co., Ltd.

1. **Preparation of samples:** Preparation of tissue samples: Take an appropriate amount of tissue samples after whole animal perfusion with 0.9% NaCl containing 0.16 mg/ml

sodium heparin. Add 100 μl of SOD Sample Preparation Solution per 10mg of tissues, and homogenize tissues at 4℃ or on ice. Centrifuge at 12,000×g at 4℃ for 3-5 minutes and take the supernatant for subsequent assay.

2. **Preparation of reagents:**

a. Preparation of WST-8/enzyme working solution: Referring to the table below, mix 151μl of SOD Assay Buffer, 8μl of WST-8 and 1μl of Enzyme Solution to obtain 160μl of WST-8/Enzyme working solution for each assay.

b. Preparation of reaction starting working solution: Thaw the Reaction Starting Solution (40X) provided in the kit and mix well before use. Prepare an appropriate amount of reaction starting working solution by diluting the Reaction Starting Solution (40X) with the SOD Assay Buffer to 1X.

3. Assay Procedures:

a. Set up reactions in a 96-well plate as follows. Mix well after adding the reaction start working solution.

	sample	blank control 1	blank control 2 bl	blank control 3*
Sample to be assayed	20 μL	—	—	20 μL
SOD Assay Buffer		20 μL	40μl	20 μL
WST-8/Enzyme working solution	160 μL	160 μL	160 μL	160 μL
reaction starting working solution	20 μL	20 μL	—	—

b. Incubate at 37℃ for 30 minutes.

c. Measure the absorbance at 450 nm.

4. Calculation of total SOD activity in samples:

a. Calculation of inhibition percentage: Inhibition percentage = [(Ablank control 1 - Ablank control 2) - (Asample - Ablank control 3)] / (Ablank control 1 - Ablank control 2) × 100%

b. Definition of SOD activity unit: when the inhibition percent in the xanthine oxidase coupling reaction system is 50%, the SOD activity in the reaction is defined as one unit.

c. Calculation of SOD enzyme activity.

3. CAT activity

First, preheat the microplate reader (Varioskan LUX)) for 30 minutes and adjust to zero with ddH_2O. Follow the detection kit provided by Beijing Solar BioScience & Technology Co., Ltd. Weigh approximately 0.1 g of tissue, add 1 mL of extraction solution, and homogenize in an ice bath. Centrifuge at 8000 g at 4°C for 10 min, and take the supernatant. Add 10 ul of supernatant and 190ul of working solution to the 96-well UV plate, mix immediately and time, record the initial absorbance value A1 at 240nm in 5s and the absorbance value A2 after 1min5s. Then calculated ⊿A=A1-A2.

CAT (U/mg prot) = 459 * ⊿A/Cpr * F (Notes: Cpr: Sample protein concentration; F: Dilution factor).

4. POD activity

First, preheat the microplate reader (Varioskan LUX)) for 30 minutes and adjust to zero with ddH_2O. Follow the detection kit provided by Beijing Solar BioScience & Technology Co., Ltd.

Weigh approximately 0.1 g of tissue, add 1 mL of extraction solution, and homogenize in an ice bath. Centrifuge at 8000 g at 4°C for 10 min, and take the supernatant. Then measure the supernatant according to the instruction manual, and record the absorbance value A1 at 30s at 470nm and the absorbance value A2 after 1min30s. A2. Then calculated ⊿A=A1-A2

POD (U/mg prot) = 7133 * ⊿A/Cpr (Notes: Cpr: Sample protein concentration; F: Dilution factor).

5. AKP activity

1. Reagent Preparation:

a. Thaw all reagents and equilibrate them to room temperature (25-28°C) prior to use.

b. To prepare Chromogenic Substrate solution, 5ml of Assay Buffer to one tube of Chromogenic Substrate.

c. Prepare a 0.5mM standard solution by diluting 10μl of p-nitrophenol Solution (10mM) in 0.2ml of Assay Buffer. Mix well and keep it in the dark.

2. Sample preparation:

a. Preparation of tissue lysate: Use the Cell Lysis Buffer to lysis tissues.

b. Preparation of plasma, serum and urine: Plasma and serum can be tested directly after preparation with conventional methods.

3. Set up reactions in 96-well plates for blank controls, standards, and samples, as indicated in the following table. Generally, add 50μLm of samples directly into sample wells. For standard solution (0.5mM), add 4, 8, 16, 24, 32, and 40μl into standard wells, respectively.

	Blank Control	Standard	Sample
Assay Buffer	50 μL	(100-x) μL	(50-y) μL
Chromogenic Substrate	50 μL	—	50 μL
Sample	—	—	y μL
Standard Solution	—	X μL	—

4. Mix well by pipetting gently.

5. Incubate at 37ºC for 5-10 minutes.

6. Add 160μl of Stop Solution into each well.

7. Measure the absorbance at OD 405nm.

8. Unit definition of AKP: One unit is defined as the amount of AKP required to hydrolyze the p-nitrophenyl phosphate to produce 1 micromole of p-nitrophenol per minute at pH 9.8 and 37ºC in diethanolamine (DEA) buffer.

9. Calculate the AKP activity in samples according to the unit definition of AKP.

6. ACP activity

1. Reagent Preparation:

a. Thaw all reagents and equilibrate them to room temperature (25-28°C) prior to use.

b. To prepare Chromogenic Substrate solution, 5ml of Assay Buffer to one tube of Chromogenic Substrate.

c. Prepare a 0.5mM standard solution by diluting 10μl of p-nitrophenol Solution (10mM) in 0.2ml of Assay Buffer. Mix well and keep it in the dark.

2. Sample preparation:

a. Preparation of tissue lysate: Use the Cell Lysis Buffer to lysis tissues.

b. Preparation of plasma, serum and urine: Plasma and serum can be tested directly after preparation with conventional methods.

3. Set up reactions in 96-well plates for blank controls, standards, and samples, as indicated in the following table. Generally, add 50μLm of samples directly into sample wells. For standard solution (0.5mM), add 4, 8, 16, 24, 32, and 40μl into standard wells, respectively.

	Blank Control	Standard	Sample

Assay Buffer	40 μL	(80-x) μL	(40-y) μL
Chromogenic Substrate	40 μL	—	40 μL
Sample	—	—	y μL
Standard Solution	—	XμL	—

4. Mix well by pipetting gently.
5. Incubate at 37ºC for 5-10 minutes.
6. Add 160μl of Stop Solution into each well.
7. Measure the absorbance at OD 405nm.
8. Unit definition of acid phosphatase activity: One unit is defined as the amount of acid phosphatase required to hydrolyzep-nitrophenyl phosphate to produce 1 micromole of p-nitrophenol in a total reaction volume of 240μl per minute at 37°C.
9. Calculate the activity of ACP in samples according to the unit definition of enzyme activity.

Supplemental Methods - qRT-PCR analysis

1.Prepare qPCR reactions:

a. Thaw and mix well the SYBR Green qPCR Mix (2X). Once melted, keep it on ice.

b. Assemble qPCR reactions on ice as follows (in a 96-well plate):

Table S1. The component and volume

Component	Volume(μL)
SYBR Green qPCR Mix (2X)	10
Forward and Reverse Primer Mix (3μM each)	2
Template DNA	2
RNase-free Water	6
Total Volume	20

c. Mix well reactions by gentle vortex or pipetting. Centrifuge briefly to collect the liquid at the bottom of the PCR tube.

2. Transfer qPCR reactions to a qPCR instrument and run thermocycling conditions as follows:

Table S2. thermocycling conditions

Step	Temperature（°C）	Duration	Cycles
Initial Denaturation	95	2 min	1
Denaturation	95	15 sec	40
Annealing and Extension	60	30 sec	
Melting Curve	95	15 sec	1
	60	15 sec	
	95	15 sec	

3. qPCR data analysis using the software provided in the qPCR instrument.
