# Supplementary material for: Effects and Mechanisms of Dufulin Toxicity on Zebrafish, Danio rerio
Source: Toxics. 2025 Dec 13;13(12):1075. doi: 10.3390/toxics13121075 (PMC12737344; doi:10.3390/toxics13121075)
Supplement: Supplementary file 1 [file toxics-13-01075-s001.zip › toxics-4036869_supplementary information-edited.pdf]

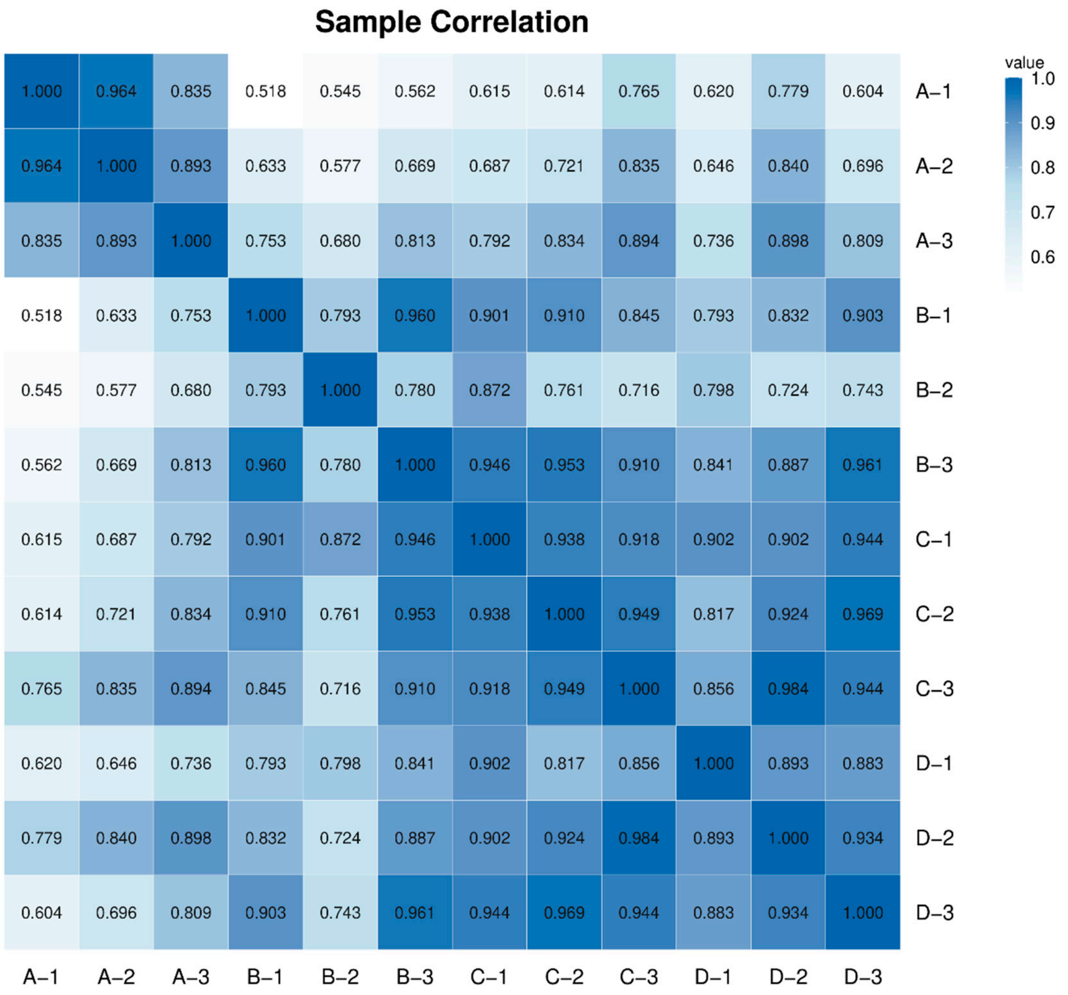

Figure S1. The Pearson correlation coefficient between samples.

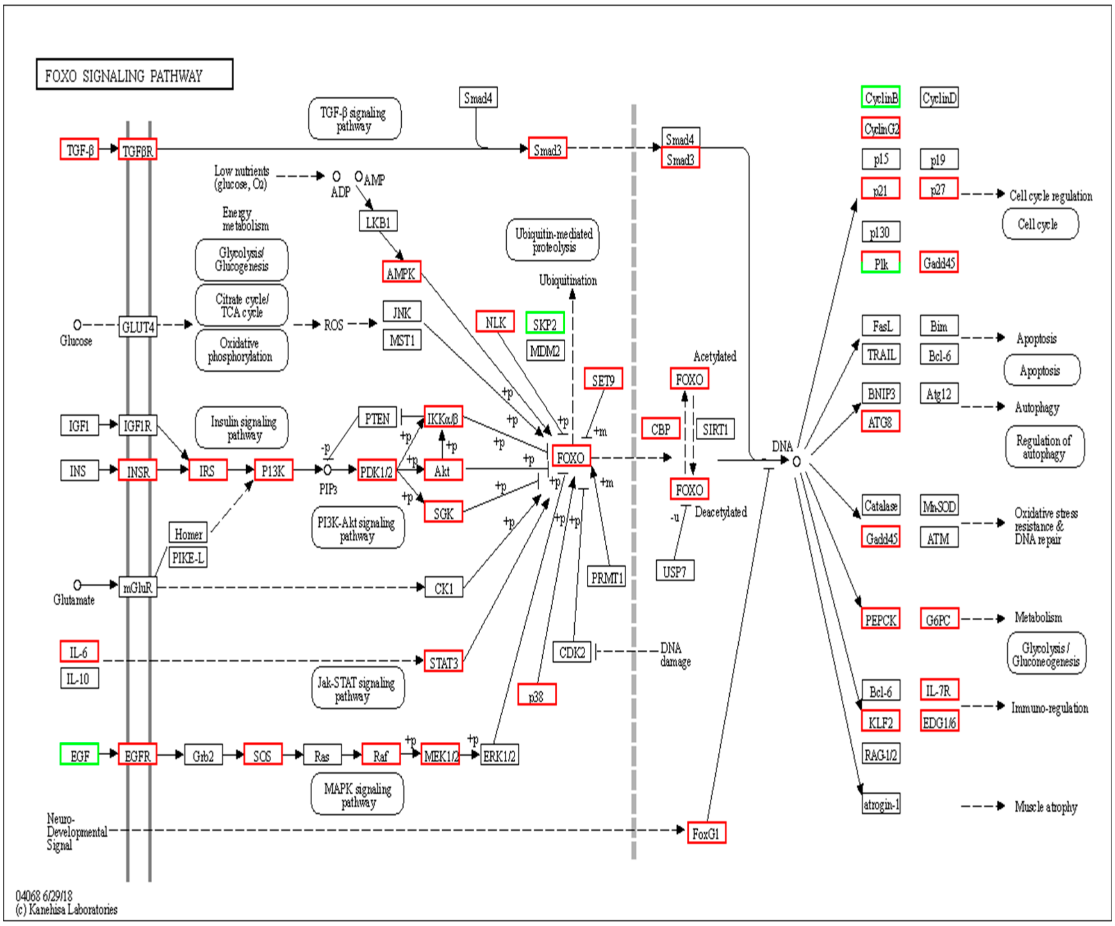

Figure S2. The most significantly enriched pathway in 0 mg/L VS 0.01 mg/L: FoxO signaling pathway.

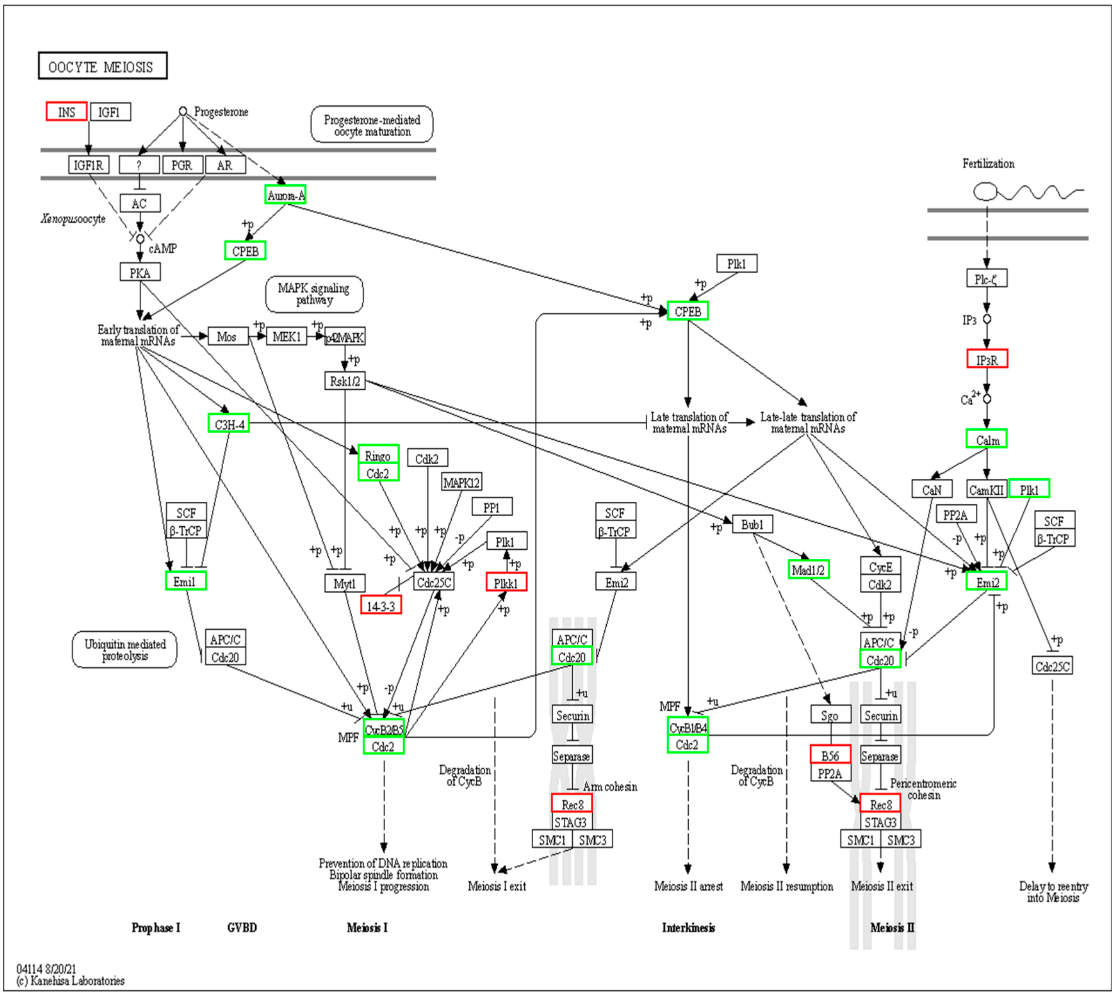

Figure S3. The most significantly enriched pathway in 0 mg/L VS 0.01 mg/L: Oocyte meiosis signaling pathway.

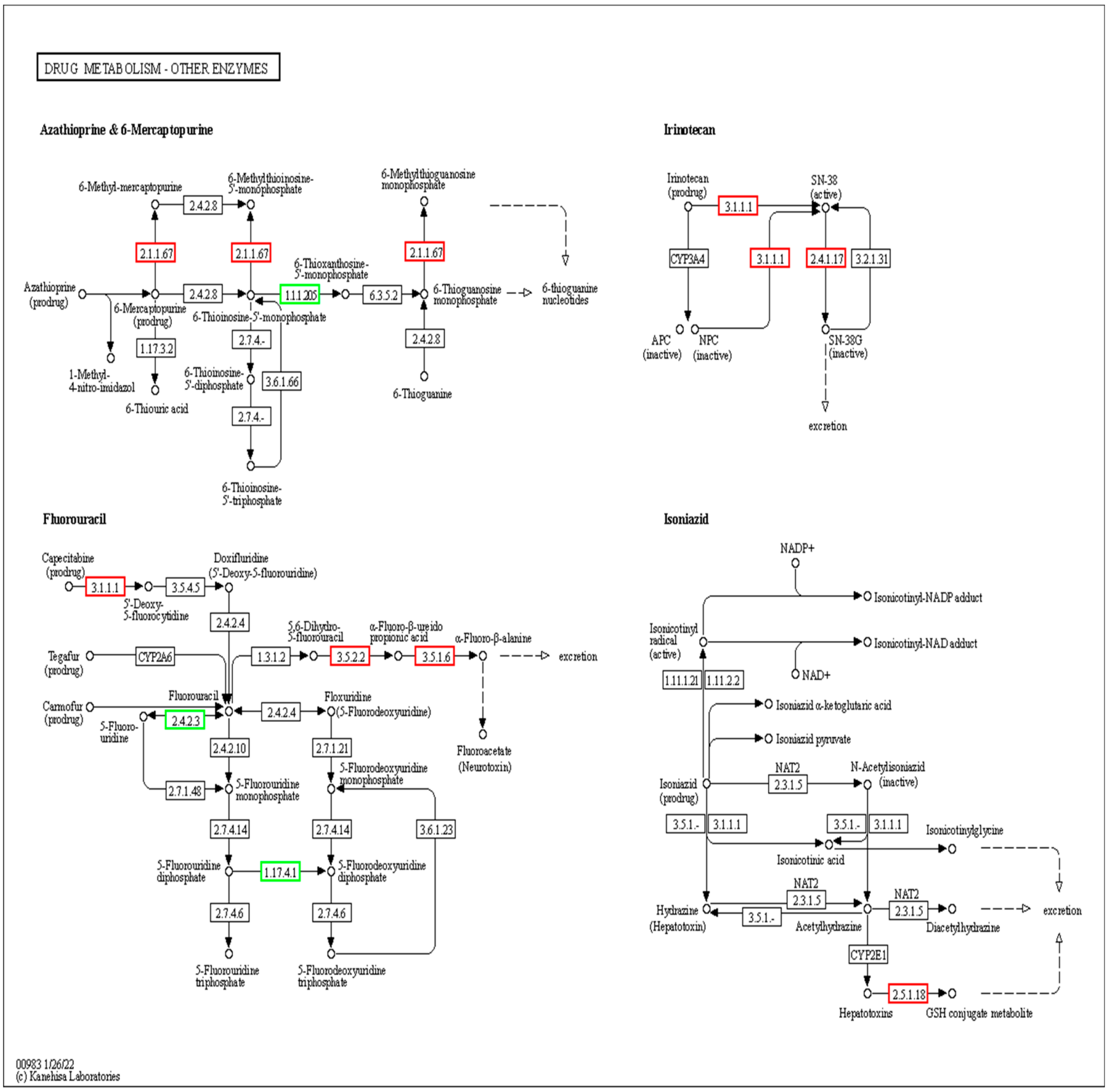

Figure S4. The most significantly enriched pathway in 0 mg/L VS 0.01 mg/L: Drug metabolism signaling pathway.
